# Cardiopulmonary exercise test to detect cardiac dysfunction from pulmonary vascular disease

**DOI:** 10.1186/s12931-024-02746-w

**Published:** 2024-03-11

**Authors:** Mona Alotaibi, Jenny Z. Yang, Demosthenes G. Papamatheakis, W. Cameron McGuire, Timothy M. Fernandes, Timothy A. Morris

**Affiliations:** Division of Pulmonary, Critical Care and Sleep Medicine, University of California, San Diego Healthcare, 200 West Arbor Drive, San Diego, CA 92103-8378 USA

**Keywords:** Cardiopulmonary exercise test (CPET), Echocardiography, Pulmonary embolism, Stroke volume augmentation, Pulmonary vascular disease

## Abstract

**Background:**

Cardiac dysfunction from pulmonary vascular disease causes characteristic findings on cardiopulmonary exercise testing (CPET). We tested the accuracy of CPET for detecting inadequate stroke volume (SV) augmentation during exercise, a pivotal manifestation of cardiac limitation in patients with pulmonary vascular disease.

**Methods:**

We reviewed patients with suspected pulmonary vascular disease in whom CPET and right heart catheterization (RHC) measurements were taken at rest and at anaerobic threshold (AT). We correlated CPET-determined O_2_·pulse_AT_/O_2_·pulse_rest_ with RHC-determined SV_AT_/SV_rest_. We evaluated the sensitivity and specificity of O_2_·pulse_AT_/O_2_·pulse_rest_ to detect SV_AT_/SV_rest_ below the lower limit of normal (LLN). For comparison, we performed similar analyses comparing echocardiographically-measured peak tricuspid regurgitant velocity (TRV_peak_) with SV_AT_/SV_rest_.

**Results:**

From July 2018 through February 2023, 83 simultaneous RHC and CPET were performed. Thirty-six studies measured O_2_·pulse and SV at rest and at AT. O_2_·pulse_AT_/O_2_·pulse_rest_ correlated highly with SV_AT_/SV_rest_ (*r* = 0.72, 95% CI 0.52, 0.85; *p* < 0.0001), whereas TRV_peak_ did not (*r* = -0.09, 95% CI -0.47, 0.33; *p* = 0.69). The AUROC to detect SV_AT_/SV_rest_ below the LLN was significantly higher for O_2_·pulse_AT_/O_2_·pulse_rest_ (0.92, SE 0.04; *p* = 0.0002) than for TRV_peak_ (0.69, SE 0.10; *p* = 0.12). O_2_·pulse_AT_/O_2_·pulse_rest_ of less than 2.6 was 92.6% sensitive (95% CI 76.6%, 98.7%) and 66.7% specific (95% CI 35.2%, 87.9%) for deficient SV_AT_/SV_rest_.

**Conclusions:**

CPET detected deficient SV augmentation more accurately than echocardiography. CPET-determined O_2_·pulse_AT_/O_2_·pulse_rest_ may have a prominent role for noninvasive screening of patients at risk for pulmonary vascular disease, such as patients with persistent dyspnea after pulmonary embolism.

## Introduction

Cardiopulmonary exercise testing (CPET) had been proposed as a noninvasive method to detect pulmonary vascular disease among patients with dyspnea and exercise intolerance after acute pulmonary embolism (PE) [1, 2] Pulmonary vascular disease-associated cardiac limitation is manifested by inadequate stroke volume (SV) augmentation in response to exercise [3, 4]. CPET has disclosed evidence of inadequate SV augmentation in over half of patients with dyspnea after PE [5]. However, CPET findings suggestive of pathologically decreased SV augmentation have never been validated against the gold standard of direct measurement by right heart catheterization (RHC) in patients with pulmonary vascular disease.

The “direct Fick method” of measuring SV by RHC requires simultaneous determination of oxygen consumption rate (VO_2_), mixed venous O_2_ content, arterial O_2_ content and heart rate. The procedure, though highly accurate, is too invasive and too expensive to evaluate stroke volume augmentation among the vast numbers of patients with post-PE dyspnea. SV, however, is related to VO_2_/heart rate (O_2_·pulse) and the difference between arterial and mixed venous oxygen content (C_a−v_O_2_) according to the equation.

$$SV{\text{ }} = {\text{ }}{O_2} \cdot pulse/{C_{a - v}}{O_2}.$$

It has been shown among patients with pulmonary vascular disease that the trajectory of O_2_·pulse increase during exercise does indeed reflect the pattern expected of SV increase [6]. Furthermore, since (C_a−v_O_2_) increases predictably between rest and anaerobic threshold (AT), SV augmentation between rest and AT (SV_AT_/SV_rest_) is reflected on CPET by the relative increase in O_2_·pulse between AT and rest (O_2_·pulse_AT_/O_2_·pulse_rest_): [7]

$$S{V_{AT}}/S{V_{rest}} = {\text{ }}\left( {{O_2} \cdot {e_{AT}}/{O_2} \cdot {e_{rest}}} \right)/\left( {{C_{a - v}}{O_{2\_AT}}/{C_{a - v}}{O_{2\_rest}}} \right).$$

We retrospectively reviewed our clinical experience with combined CPET and RHC examinations to determine whether, in patients with suspected pulmonary vascular disease, O_2_·pulse_AT_/O_2_·pulse_rest_, measured noninvasively by CPET predicts abnormally low SV_AT_/SV_rest_, measured invasively by RHC.

## Methods

### Subjects

We reviewed the results of simultaneous RHC and CPET among consecutive patients suspected of having pulmonary vascular disease who were referred to the University of California, San Diego from January 2018 through February 2023. RHC-CPET was performed based on the clinical judgement of the pulmonary vascular specialist. Among patients with more than one study, we evaluated only the first study. Inclusion criteria were: (1) measurement, at rest, of heart rate (HR_rest_), cardiac output (Q_rest_) by the direct Fick method and O_2_·pulse_rest_; (2) measurement, at an independently determined AT point, of O_2_·pulse_AT_; and (3) measurement, when VO_2_ was within 20% of the VO_2_ at AT, of HR_AT_ and Q_AT_. There were no exclusion criteria. The University of California, San Diego Institutional Review Board approved the study (IRB #171,888).

### Right heart catheterization and SVA_AT_

RHC was performed at rest and during exercise as previously described [8]. A radial artery catheter and a pulmonary artery catheter were inserted in the cardiac catheterization laboratory while patients were in the supine position. Right atrial, right ventricular, pulmonary artery, and pulmonary artery occlusion pressure were measured in succession. Once a stable respiratory quotient was observed with the patient at rest, heart rate was recorded, and cardiac output was determined with the direct Fick method from the measured VO2 and simultaneous radial artery and pulmonary artery blood gases.

The subjects then performed incrementally increased exercise on a supine cycle ergometer (Medical Positioning, Inc.), as described below. Heart rate and cardiac output measurements were repeated in a similar fashion during exercise at point near anerobic threshold (as determined in real-time by a change in the slope VCO2 versus VCO2) and again near peak exertion.

Hemodynamic data were collected without knowledge of the CPET results. SV_AT_/SV_rest_ was calculated as.

$$S{V_{AT}}/S{V_{rest}} = {\text{ }}\left( {{Q_{AT}}/{\text{ }}H{R_{AT}}} \right){\text{ }}/{\text{ }}\left( {{Q_{rest}}/{\text{ }}H{R_{rest}}} \right),$$

where Q_AT_ and Q_rest_ represent cardiac output at anaerobic threshold and at rest, respectively, and HR_AT_ and HR_rest_ represent heart rate at anaerobic threshold and at rest, respectively. We pre-specified the lower limit of normal for SV_AT_/SV_rest_ based right heart catheterization data from healthy volunteers, in whom stroke volume increased by 38.8% (SD 5.2%) between rest and AT [7]. We arbitrarily selected the mean minus two times the standard deviation from that experiment (128%) as the SV_AT_/SV_rest_ lower limit of normal (LLN) for the current study.

### Cardiopulmonary exercise test determination of O_2_·pulse_rest_ and O2·pulse_AT_

**Fig. 1:**
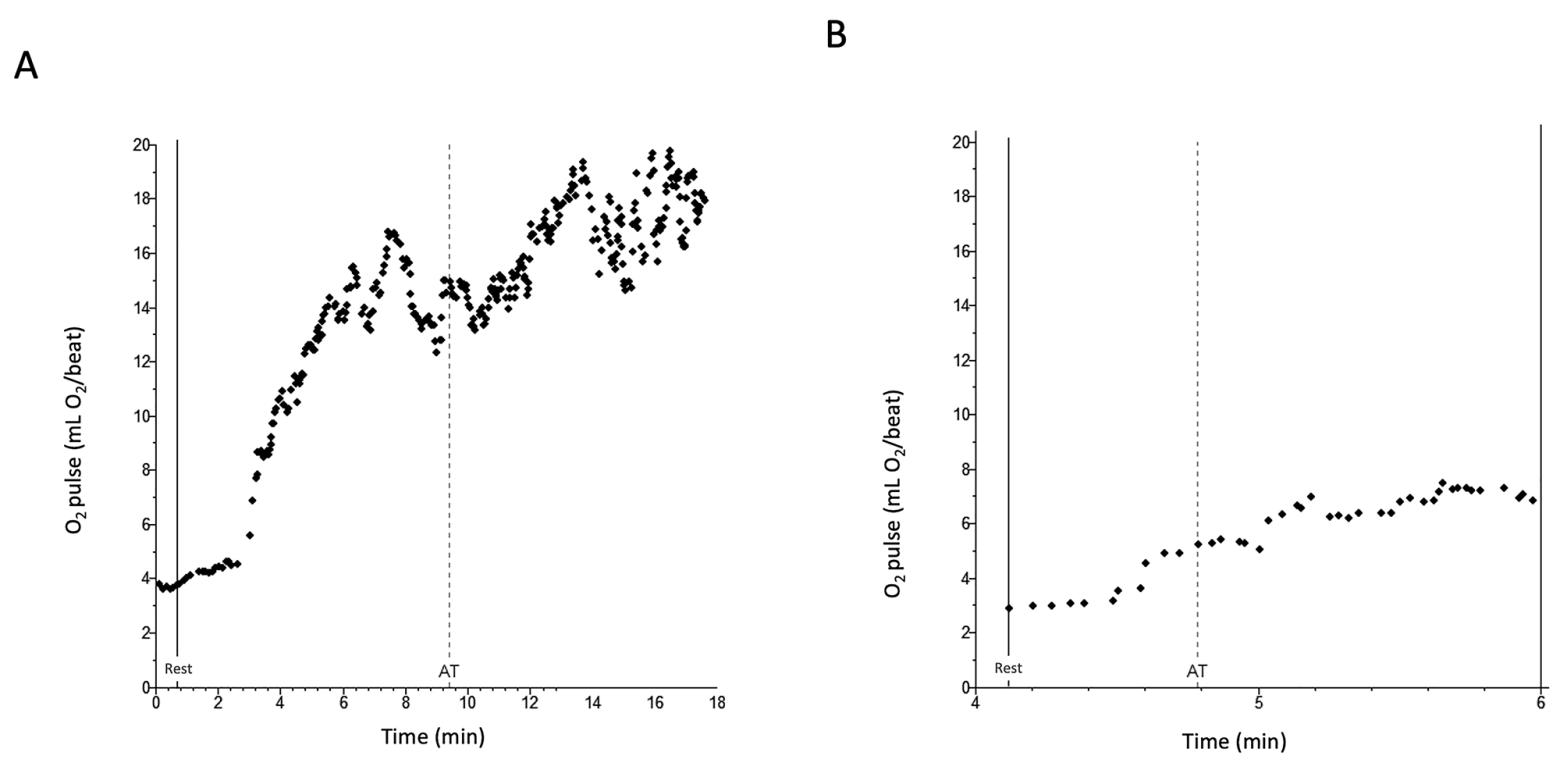
O_2_·pulse at rest (O_2_·pulse_rest_) and at anaerobic threshold (O_2_·pulse_AT_). **(A)** Normal increase in O_2_·pulse from rest (O_2_·pulse_rest_, solid vertical line) to anaerobic threshold (O_2_·pulse_AT_, dashed vertical line). O_2_·pulse_rest_ was 4.5 ml O_2_/beat and O_2_·pulse_AT_ was 14.5 ml O_2_/beat. **(B)** Pathologically low increase from O_2_·pulse_rest_ to O_2_·pulse_AT_. O_2_·pulse_rest_ was 3.8 ml O_2_/beat and O_2_·pulse_AT_ was 7.3 ml O_2_/beat

Simultaneously with the RHC, we performed incremental symptom-limited CPET with the patients on a recumbent bicycle, using a stepwise exercise protocol to produce a uniform increase in work rate and metabolic energy expenditure per incremental step. O_2_·pulse_rest_ was determined during steady state rest with a V-Max metabolic cart (CareFusion, San Diego, CA) or a Ultima Cardio2 metabolic cart (MGC Diagnostics, St Paul, MN) from VO_2_, measured through breath-by-breath analysis of inspired and expired gases and heart rate, measured by continuous electrocardiography (Fig. 1).

Anaerobic threshold was determined by a board-certified pulmonologist (TM) without knowledge of the RHC results through analysis of the slopes of VCO_2_ vs. VO_2_ (V-slope) as well as VE/VO_2_, VE/VCO_2_, P_ET_O_2_ and P_ET_CO_2_ vs. time, with the V-slope method as the decisive criteria. O_2_·pulse_AT_ was determined retrospectively during the 20-second interval containing the anaerobic threshold point (Fig. 1).

### Echocardiography

For comparison, peak tricuspid regurgitation velocity (TRV_peak_) and other signs of pulmonary vascular disease [9] were recorded from echocardiograms that had been performed within six months of the combined CPET-RHC studies. In addition, the echocardiographic results were categorized as high- or intermediate-risk vs. low-risk, according to the recommendations of the European Society of Cardiology and the European Respiratory Society (ESC/ERS) Task Force for the Diagnosis and Management of Acute Pulmonary Embolism [10] and the ESC/ERS Task Force for the Diagnosis and Treatment of Pulmonary Hypertension [9].

### Statistical analysis

Continuous variables are presented as mean (+/- standard deviation) or median and interquartile range [IQR]. Categorical variables are presented as number (%). The Shapiro-Wilk test was used to evaluate SV_AT_/SV_rest_ values, accepting *p* > 0.05 as confirmation of their normal distribution. Pearson correlation was used to compare O_2_·pulse_AT_/O_2_·pulse_rest_ to SV_AT_/SV_rest_ and to compare TRV_peak_ to SV_AT_/SV_rest_. O_2_·pulse_AT_/O_2_·pulse_rest_ and TRV_peak_ were also linearly regressed on SV_AT_/SV_rest_. Receiver operating characteristic curves were plotted to compare the sensitivities and specificities of O_2_·pulse_AT_/O_2_·pulse_rest_ and TRV_peak_ for detecting SV_AT_/SV_rest_ below the lower limit of normal (LLN = 1.28) [7]. We pre-defined 90% as an acceptable sensitivity for a screening test to detect SV_AT_/SV_rest_ below the LLN based the consensus of clinical judgment within our research team. Statistical calculations were performed with Prism version 9 (GraphPad Software, San Diego CA).

## Results

### Study population

**Fig. 2:**
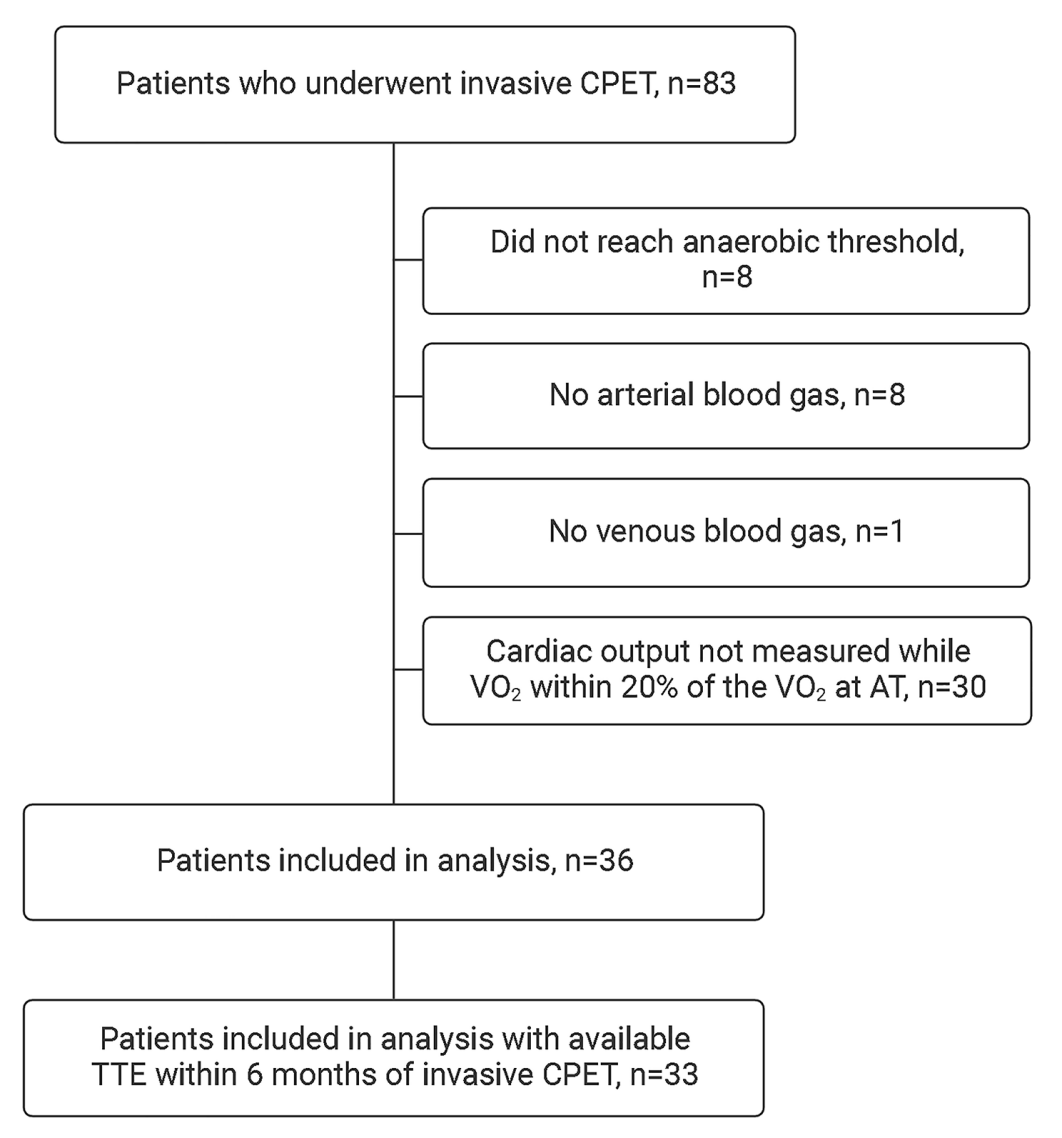
Patient selection. CPET, cardiopulmonary exercise test; VO_2_, oxygen consumption; AT, anaerobic threshold; TTE, transthoracic echocardiography

During the study period, 83 simultaneous RHC and CPET tests were performed. Forty-seven tests were not included because blood was not sampled from the systemic artery (*n* = 8) or pulmonary artery (*n* = 1) for direct Fick cardiac output measurement; anaerobic threshold was not reached or was indeterminate (*n* = 8); or cardiac output was not measured during exercise while the VO_2_ was within 20% of VO_2_ at anaerobic threshold (*n* = 30). Thirty-six tests met the inclusion criteria and were included in the analysis (Fig. 2). The demographics and hemodynamics of the excluded patients were not different from the included patients (Table e1).

Included patients were 56.5 [40.25, 66] years of age and had body mass index (BMI) of 28 [24.1, 32.5] kg/m^2^ (Table 1). There were 11 (30.6%) men and 25 (69.4%) women. 12 patients (33.3%) had co-existing cardiopulmonary comorbidities. All patients exercised to the point of volitional exhaustion, as communicated directly to the test operators, without adverse effects. All patients reached anaerobic threshold and none demonstrated a plateau in O_2_·pulse, V_E_ or VO_2_ over time prior to the points at which we collected data for the study. The distribution of hemodynamic measurements and CPET parameters are illustrated in Tables 2 and 3, respectively. The Shapiro–Wilk test supported that SV_AT_/SV_rest_ data were normally distributed (W = 0.9539, *p* = 0.1388).

**Table 1:** Patient Characteristics (*n* = 36) Data are presented as median [interquartile range] or count (percent) unless otherwise indicated

Patient Characteristics mn1	Included
Age, years, median [IQR]	56.5 [40.25, 66]
Male	11 (30.6)
BMI, kg/m^2^	28 [24.1, 32.5]
Final Diagnosis	
Pulmonary Arterial Hypertension	8 (22.2)
Lung disease	1 (2.8)
Left Ventricular failure	5 (13.9)
CTED/CTEPH	11 (30.6)
Other	11 (30.6)
History of Pulmonary embolism	18 (50.0)
Coexisting cardiovascular disease	12 (33.3)
Coexisting cardiovascular disease type	
Coronary artery disease	2/12 (16.7)
Hypertension	10/12 (83.3)
Coexisting lung disease	12 (33.3)
Coexisting lung disease type	
Asthma	4/12 (36.4)
Diffuse parenchymal lung disease	4/12 (36.4)
Obstructive sleep apnea	4/12 (36.4)

BMI, body mass index. CTED, chronic thromboembolic disease. CTEPH, chronic thromboembolic pulmonary hypertension

**Table 2:** Hemodynamic Parameters at rest, anerobic threshold and peak exercise. Data are presented as median [interquartile range (IQR)] unless otherwise indicated. *Cardiac output was calculated by the direct Fick method

	Rest	Anerobic threshold	Peak exercise
mean arterial blood pressure, mmHg	100.5 [92, 108]	118.5 [112, 126]	128 [116, 133]
mean PAP, mmHg	19 [15, 23]	32 [22.8, 34.8]	38 [30.5, 42.5]
PAOP, mmHg	12 [10, 14]	18 [15.3, 20]	22 [18, 26]
PVR, Wood Units	1.1 [0.5, 2.0]	1.068 [0.69, 1.67]	0.95 [0.7, 1.5]
Cardiac output, L/min	6 [5, 8]	10.2 [9.0, 13.4]	13.2 [11.3, 15.9]
Cardiac index, L/min/m2	3 [3, 4]	5.9 [4.4, 7.1]	6.8 [6.1, 7.9]
Stroke volume, ml/beat	91.4 [65.4, 122.7]	96.2 [85.7, 124.8]	98.1 [84.7, 120.8]
PaO_2_, mmHg	86 [78, 97]	79 [72, 95.5]	79 [72.5, 91.3]
PaCO_2_, mmHg	38 [34, 40]	38 [35.3, 41.5]	36 [33, 40]
PvO_2_, mmHg	39 [36, 43]	30.5 [27.2, 33]	28 [24, 30]
Arterial saturation, %	98 [97, 99]	97.4 [95.4, 98.6]	97.6 [95.2, 98.6]
PA saturation, %	76 [71, 79]	56.8 [50.1, 59.1]	46.7 [38.9, 51.3]

PAP, pulmonary artery pressure. PAOP, pulmonary artery occlusion pressure. PVR, pulmonary vascular resistance. PaO2, partial pressure of oxygen. PaCO2, partial pressure of carbon dioxide. PvO2, mixed venous oxygen pressure. PA, pulmonary artery

**Table 3:** Cardiopulmonary exercise test parameters data are presented as median [interquartile range (IQR)] unless otherwise indicated

	Rest	Anerobic Threshold	Peak Exercise
Work, watts	0	10.00 [0.00, 42.50]	75.00 [37.50, 97.50]
VO2, L/min	0.3 [0.2, 0.3]	0.8 [0.7, 0.9]	1.1 [1.01, 1.5]
VO2/wt, ml/kg/min	3.2 [2.8, 4.1]	10.4 [8.9, 12.4]	15.1 [13.3, 19.5]
O2 pulse, ml/beat	3.54 [3.12, 4.46]	8.30 [6.75, 9.00]	10.13[7.84, 11.41]
VE, L/min	9.20 [8.30, 10.25]	23.35 [18.46, 32.44]	44.50 [35.25, 61.75]
VE/VCO2	39.43 [36.18, 43.58]	32.00 [30.00, 36.00]	32.37 [30.50, 39.78]
VE/VCO2 slope	29.25 [27.08, 33.77]		
RER	0.87 [0.83, 0.90]	0.87 [0.74, 0.95]	1.07 [1.02, 1.11]
Breathing reserve, %	-	-	63.00 [53.30, 75.50]
SaO_2_, %	98 [97, 98]	98 [95, 100]	97 [96, 99]
Heart Rate, BPM	75 [65, 81]	109 [96.5,116]	139.5 [115.2, 147.8]

VO_2_, O_2_ consumption; SaO_2_, oxygen saturation

Figure legend

### Relationship between O_2_·pulse_AT_/O_2_·pulse_rest_ and SVA_AT_

**Fig. 3:**
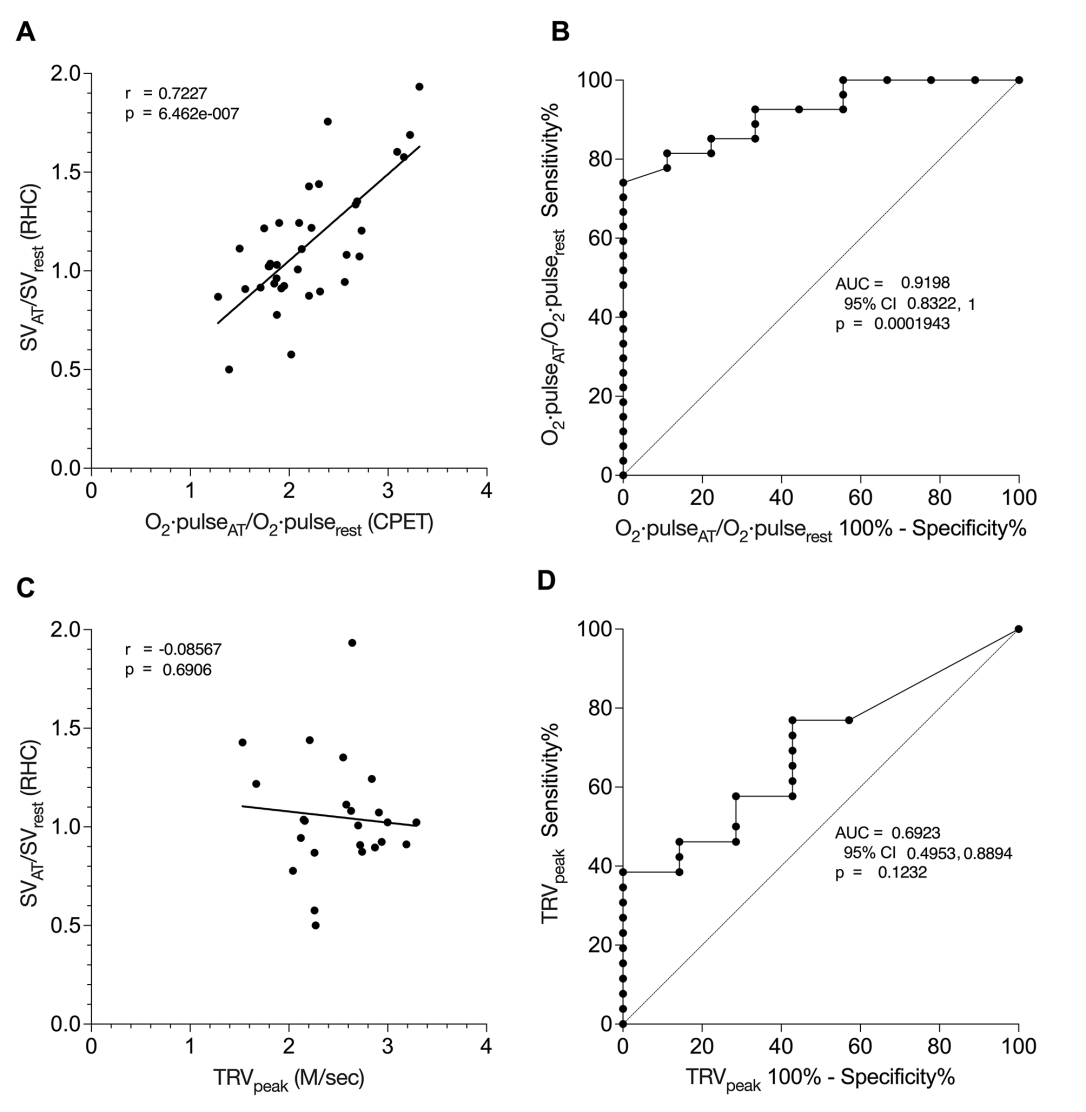
O_2_·pulse_AT_/O_2_·pulse_rest_ compared to TRV_peak_ to predict stroke volume augmentation. **A**. O_2_·pulse_AT_/O_2_·pulse_rest_ determined from CPET data (see text) correlated with stroke volume augmentation from rest to anaerobic threshold (SVA_AT_) measured by right heart catheterization (*p* < 0.0001). **B.** The ROC curve of O_2_·pulse_AT_/O_2_·pulse_rest_ to detect SVA_AT_ below the lower limit of normal (LLN) had an area under the ROC curve of 0.92 (SE 0.04, *p* = 0.0002). **C.** Tricuspid regurgitant velocity peak (TRV_peak_) measured by echocardiography did not correlate with SVA_AT_ (*p* = 0.69). **D.** The ROC curve of TRV_peak_ to detect SVA_AT_ below the LLN had an area of 0.69 (SE 0.10), which was not statistically significant (*p* = 0.12)

Figure 3A illustrates a statistically significant linear correlation between O_2_·pulse_AT_/O_2_·pulse_rest_ and SV_AT_/SV_rest_ (*r* = 0.72, 95% CI 0.52, 0.85; *p* < 0.0001). Linear regression yielded a slope of 0.51 (95% CI 0.48, 0.55) between SV_AT_/SV_rest_ and O_2_·pulse_AT_/O_2_·pulse_rest_. The LLN for SV_AT_/SV_rest_ (1.28) corresponded to O_2_·pulse_AT_/O_2_·pulse_rest_ of 2.5 (95% CI 2.3, 2.7). In contrast, Fig. 3C shows no significant correlation between TRV_peak_ and SV_AT_/SV_rest_ (*r* = -0.09, 95% CI -0.47, 0.33; *p* = 0.69).

The area under the receiver operating characteristic curve (AUROC) of O_2_·pulse_AT_/O_2_·pulse_rest_ for detecting SV_AT_/SV_rest_ below the LLN (Fig. 3B) was 0.92 (SE 0.04, *p* = 0.0002). The AUROC 95% CI was 0.832 to 1.00. O_2_·pulse_AT_/O_2_·pulse_rest_ of less than 2.6 was 92.6% sensitive (95% CI 76.6%, 98.7%) and 66.7% specific (95% CI 35.2%, 87.9%). O_2_·pulse_AT_/O_2_·pulse_rest_ of less than 2.2 was only 74% sensitive (95% CI 55.3%, 86.8%) but 100% specific (95% CI 70.1%, 100%).

The AUROC of TRV_peak_ to detect SV_AT_/SV_rest_ below the LLN (Fig. 3D) was 0.69 (SE 0.10), which was not statistically significant (*p* = 0.12). The AUROC 95% CI was 0.495 to 0.889, which includes the nondiscriminatory value of 0.5. No value of TRV_peak_ had a sensitivity of 90% or higher. Echocardiography meeting the ESC/ERS criteria [10] for intermediate- or high-risk (TRV_peak_ >2.8 and/or presence of two other pulmonary hypertension signs [9]) was only 23.0% sensitive (95% CI 11.0%, 42.0%) but 100% specific (95% CI 64.6%, 100%) for detecting SV_AT_/SV_rest_ below the LLN.

## Discussion

We validated the accuracy of O_2_·pulse_AT_/O_2_·pulse_rest_ to predict SV_AT_/SV_rest_ in a consecutive series of patients receiving combined CPET and RHC for the clinical evaluation of dyspnea potentially related to pulmonary vascular disease. We derived a cutoff of 2.6 for O_2_·pulse_AT_/O_2_·pulse_rest_ to predict SV_AT_/SV_rest_ less than the LLN. We observed a highly significant linear relationship between O_2_·pulse_AT_/O_2_·pulse_rest_ and SV_AT_/SV_rest_, with a slope that corresponds with previous CPET-RHC comparisons among healthy subjects and among patients with various severities of heart failure [7, 11–15]. Although O_2_·pulse_AT_/O_2_·pulse_rest_ enables merely an estimate of the RHC measurement of SV_AT_/SV_rest_, the correlation between the two is comparable to or even superior to the correlation between different invasive methods of measuring stroke volume by RHC [16–19].

SV augmentation is an important adaptation to exercise that helps increase cardiac output and maintain organ perfusion during increased oxygen utilization [12]. Increased venous pressure during exercise enhances right ventricular end-diastolic volume [20] and normally improves contractility [21]. SV rises incrementally as exercise proceeds and reaches a plateau near AT [7, 12, 22–24]. In healthy persons, there is an approximately 40% increase in SV by the time AT is reached [7]. The advantage to considering SV_AT_/SV_rest_, rather than SV_AT_ alone, is that the ratio controls for demographic factors (body size, age, sex, etc.) that typically influence SV.

Pulmonary vascular disease leads to elevated right ventricular end-diastolic volume and impaired contractility at rest, which impedes the normal adaptation to exercise [21]. As a result, SV augmentation is markedly decreased [3, 4] Residual pulmonary vascular obstruction limits SV augmentation, increases pulmonary artery resistance and compromises right ventricular function [25, 26]. Since SV augmentation substantially improves among CTEPH patients after pulmonary artery thromboendarterectomy, it is reasonable to attribute the defect to pulmonary vascular obstruction itself [4]. Furthermore, insufficient SVA in response to exercise predicts mortality from pulmonary hypertension more accurately than any other exercise parameter and enhances the accuracy of mortality prediction above the six minute walking distance alone [27].

Although CTEPH is present in only a small fraction of patients with dyspnea after PE [28], less severe pulmonary vascular disease causes respiratory symptoms [29], hypoxemia [30–32], gas exchange deficits [31, 33, 34] and exercise intolerance [35]. Residual pulmonary vascular obstruction is associated with the risk of progression to CTEPH [36]. In our previous series of CPET for patients with long-term dyspnea after acute pulmonary embolism, low O_2_·pulse_AT_/O_2_·pulse_rest_ corresponded to residual pulmonary artery obstruction [5].

Among symptomatic post-pulmonary embolism patients, low O_2_·pulse_AT_/O_2_·pulse_rest_ measured noninvasively during CPET suggests inadequate SV augmentation because of residual pulmonary vascular occlusion [5]. Validation that O_2_·pulse_AT_/O_2_·pulse_rest_ accurately reflects SV augmentation enables CPET to be an informative and practical noninvasive tool to help distinguish between pulmonary vascular disease and deconditioning or anxiety (in the absence of physiological defects) among the large number of patients with dyspnea after pulmonary embolism [1].

Our results compliment the results of Held et al. and of McCabe et al., who disclosed abnormal CPET findings in a majority of patients in whom CTED or CTEPH had been confirmed by RHC [37, 38]. As was the case in our study, echocardiography (including TRV_peak_) was unable to detect pulmonary vascular disease in 31% of CTEPH patients [37]. The insensitivity likely refects the fact that echocardiography is routinely performed at rest, which may not reflect defects that are manifested only during exercise. However, we recognize that echocardiography typically preceded RHC-CPET, at times by several months. It is possible that the difference between echocardiographic and CPET results were influenced by disease progression among some patients.

Notably, CPET data from CTED and CTEPH patients reported by Held et al. and McCabe et al. reflected ventilatory inefficiency, presumably based on ventilation/perfusion mismatching [37, 38]. We observed similar ventilatory inefficiency in our patients, which we are investigating in a separate study. The current study focuses on the ability of O_2_·pulse_AT_/O_2_·pulse_rest_, to reflect stroke volume augmentation itself. However, we anticipate that both factors are likely implicated in pulmonary vascular disease after acute PE [5, 39].

Since acute pulmonary embolism occurs in about 63/100,000 persons per year [40], up to half of whom report chronic dyspnea [41–43], the method we validated could detect SV augmentation limitation due to pulmonary vascular disease in a large number of at-risk patients [28]. It is more practical than RHC and more sensitive than echocardiography. Since our patients ranged from normal to very poor cardiopulmonary reserve during exercise, our results suggest that O_2_·pulse_AT_/O_2_·pulse_rest_ would reflect SV augmentation across a wide spectrum of dysfunction.

Besides acute pulmonary embolism, there are numerous risk factors associated with pulmonary vascular disease, including scleroderma and other connective tissue diseases. In these at-risk patients, symptoms begin with dyspnea on exertion, but pulmonary hypertension may not be present at rest. Non-invasive CPET to screen for decreased exercise-related stroke volume augmentation by detecting impaired O_2_·pulse_AT_/O_2_·pulse_rest_ has the potential to identify these patients as well, earlier in their disease course.

ESC/ERS guidelines recommend transthoracic echocardiography as an initial test to evaluate dyspnea on exertion after pulmonary embolism [10]. However, while TTE can be useful as a screen for chronic thromboembolic pulmonary hypertension (CTEPH), it may not be the best approach for evaluating patients who are limited by persistent perfusion defects that cause exercise-induced pulmonary hypertension. Our present research has shown that tricuspid regurgitant velocity peak is less sensitive than non-invasively measured O2·pulse_AT_/O2·pulse_rest_ in detecting directly measured SVA AT below the lower limit of normal. Therefore, we recommend non-invasive cardiopulmonary exercise testing (CPET) as the first step in evaluating dyspnea after PE.

Like other studies of CPET and RHC for pulmonary vascular disease [37, 38], our study is limited by its relatively small size and its retrospective nature. In addition, because O_2_·pulse_AT_/O_2_·pulse_rest_, reflects SV augmentation at AT, our study included only RHC tests that measured both SV_rest_ and SV near AT. Estimation of SV augmentation from O_2_·pulse ratios at other times would have been erroneous due to changes in oxygen extraction and heart rate during exercise above AT [7, 12, 22–24]. Nevertheless, with careful attention to technique, SV_AT_/SV_rest_ could serve as a standard by which to evaluate SV augmentation during exercise.

A limitation of our study is that we selected the LLN for SV_AT_/SV_rest_ based on RHC-CPET studies performed on young, healthy subjects during upright cycling. Supine position increases venous return at rest and may lower the relative increase in diastolic volume during exercise that contributes to the SV response [20]. Further studies are needed to determine if the predicted and LLN for SV_AT_/SV_rest_ should be different between upright and recumbent CPETs.

Chronotropic incompetence may confound the clinical implication of stroke volume augmentation, since slow heart rates during exercise would allow more time for diastolic filling and potentially dampen the effect of cardiac dysfunction on stroke volume during exercise. For example, among the five patients in our study who were taking beta blockers, pharmacological slowing of the heart rate, rather than inherent recovery of the ventricles, might have lead to increased stroke volume during exercise. Nevertheless, since beta blockers are unlikely to change C_a−v_O_2_AT_/C_a−v_O_2_rest_, we reasoned that it was still informative to include those patients in the validation that stroke volume augmentation is reflected by the O_2_·pulse_AT_/O_2_·pulse_rest_.

However, it is unlikely that chronotropic incompetence played a substantial role in the current study, since heart rates were similar between subjects with normal SVA (110.3 +/- 19.5) and those with low SVA (107.2 +/- 17.4).

The CPET-based estimation of SV_AT_/SV_rest_ that we validated during right heart catheterization may be useful as a stand-alone test in other settings. For example, the method may be used for the noninvasive screening of ambulatory patients with a variety of cardiac and pulmonary disorders for exercise-related heart dysfunction. The method would help quantify cardiac adaptation to exercise in patients with known or suspected heart failure.

We speculate that deficient stroke volume augmentation from various types of heart failure (right side or left side, systolic or diastolic) will have similar increases in C_a−v_O_2_ between rest and AT and therefore similar effects of SV_AT_/SV_rest_ on O2·pulse_AT_/O2·pulse_rest_ [7]. However, we would not expect O2·pulse_AT_/O2·pulse_rest_ to reflect SV_AT_/SV_rest_ accurately among patients with myopathies that cause poor O2 extraction (e.g. mitochondrial enzyme defects), since the C_a−v_O_2_ might not change in a predictable fashion at AT. Although the current study did not include such patients, it is possible that myopathies could be differentiated from stroke volume augmentation defects by differences in O2·pulse trajectories subsequent to AT. Degani-Costa et al. reported flattening of the O2·pulse trajectory (and upward deflection of the heart rate vs. VO2 plot) during the later portions of exercise among subjects with pulmonary hypertension but not among those with mitochondrial myopathies [6].

We hope that our results will open a line of investigation about the role of this technique in the management of patients at risk of right ventricular dysfunction from pulmonary vascular disease. The technique may be particularly helpful in patients in whom dysfunction occurs during exercise but is not apparent at rest. Also, because SV_AT_/SV_rest_ reflects overall cardiac adaptation to exercise, it is a potential predictor of poor outcomes that may supplement the roles of peak VO_2_, VO_2_ at AT and V_E_/VCO_2_. Future research would be needed to ascertain the clinical utility of using O2·pulse_AT_/O2·pulse_rest_ to evaluate patients with dyspnea of unknown origin.

### Summary

We validated the accuracy of O_2_·pulse_AT_/O_2_·pulse_rest_, measured noninvasively by CPET, compared to invasive measurement of SV_AT_/SV_rest_ during RHC. We derived a cutoff value of 2.6, which should be validated in future studies. Our results suggest that CPET can be used to evaluate SV augmentation in symptomatic patients at risk for pulmonary vascular disease, such as those with dyspnea after acute pulmonary embolism, to detect early compromise of the right ventricle.

## Data Availability

Deidentified data relevant to this study is available upon request to the corresponding author.

## Abbreviations

AT: Anaerobic threshold
CPET: Cardiopulmonary exercise test
HR_AT_: Heart rate at anaerobic threshold
HR_rest_: Heart rate at rest
Q_AT_: Cardiac output at anaerobic threshold
Q_rest_: Cardiac output at rest
RHC: Right heart catheterization
SVA: Stroke volume augmentation
SVA_AT_: Stroke volume augmentation at anaerobic threshold measured by RHC
O_2_·pulse: Oxygen consumption rate divided by heart rate
O_2_·pulse_rest_: O_2_·pulse at rest
O_2_·pulse_AT_: O_2_·pulse at anaerobic threshold
